# Helicopter emergency medical services in major incident management: A national Norwegian cross-sectional survey

**DOI:** 10.1371/journal.pone.0171436

**Published:** 2017-02-13

**Authors:** Anne Siri Johnsen, Stephen J. M. Sollid, Trond Vigerust, Morten Jystad, Marius Rehn

**Affiliations:** 1 Department of Research and Development, Norwegian Air Ambulance Foundation, Drøbak, Norway; 2 Department of Health Studies, Faculty of Social Sciences, University of Stavanger, Stavanger, Norway; 3 Department of Anaesthesiology, Division of Emergencies and Critical Care, Oslo University Hospital, Oslo, Norway; 4 Air Ambulance Department, Oslo University Hospital, Oslo, Norway; 5 Norwegian Air Ambulance Ltd, Lørenskog/Dombås Base, Lørenskog/Dombås, Norway; Yokohama City University, JAPAN

## Abstract

**Objective:**

Helicopter Emergency Medical Services (HEMS) aim to bring a highly specialised crew to the scene of major incidents for triage, treatment and transport. We aim to describe experiences made by HEMS in Norway in the management of major incidents.

**Design:**

Doctors, rescue paramedics and pilots working in Norwegian HEMS and Search and Rescue Helicopters (SAR) January 1^st^ 2015 were invited to a cross-sectional study on experiences, preparedness and training in major incident management.

**Results:**

We identified a total of 329 Norwegian crewmembers of which 229 (70%) responded; doctors 101/150, (67%), rescue paramedics 64/78 (82%), pilots 64/101, (63%). HEMS and SAR crewmembers had experience from a median of 2 (interquartile range 0–6) major incidents. Road traffic incidents were the most frequent mechanism and blunt trauma the dominating injury. HEMS mainly contributed with triage, treatment and transport. Communication with other emergency services prior to arrival was described as bad, but good to excellent when cooperating on scene. The respondents called for more interdisciplinary exercises.

**Conclusion:**

HEMS and SAR crewmembers have limited exposure to major incident management. Interdisciplinary training on frequent scenarios with focus on cooperation and communication is called for.

## Introduction

Major incidents (MI) constitutes a major global public health problem affecting both urban and rural areas. [1–3] The definition of MI in the literature is heterogeneous, but has been referred to as an incident that requires mobilization of extraordinary emergency medical service (EMS) resources and that has been identified as a MI in that system. [4] The capacity to manage MI varies depending on type of incidents, local resources and systems. Normally, MI triggers the activation of the local health systems emergency plans. Even in high income countries where the health systems are normally robust, MI can constitute a challenge beyond the system capacity. [5] In the period between 1970–2003 a total of 80 MIs claimed 1174 lives in Norway. The incidents mainly pertained to transportation, industry, offshore activity as well as major avalanches. [6]

Helicopter emergency medical service (HEMS) and search and rescue (SAR) helicopters contribute to major incident management with transportation of equipment, personnel, and patients as well as providing overhead surveillance and perform search and rescue. [7] Although HEMS and SAR units are included in most major incident management plans, optimal utilization of this limited resource remains undecided.

**Fig 1:**
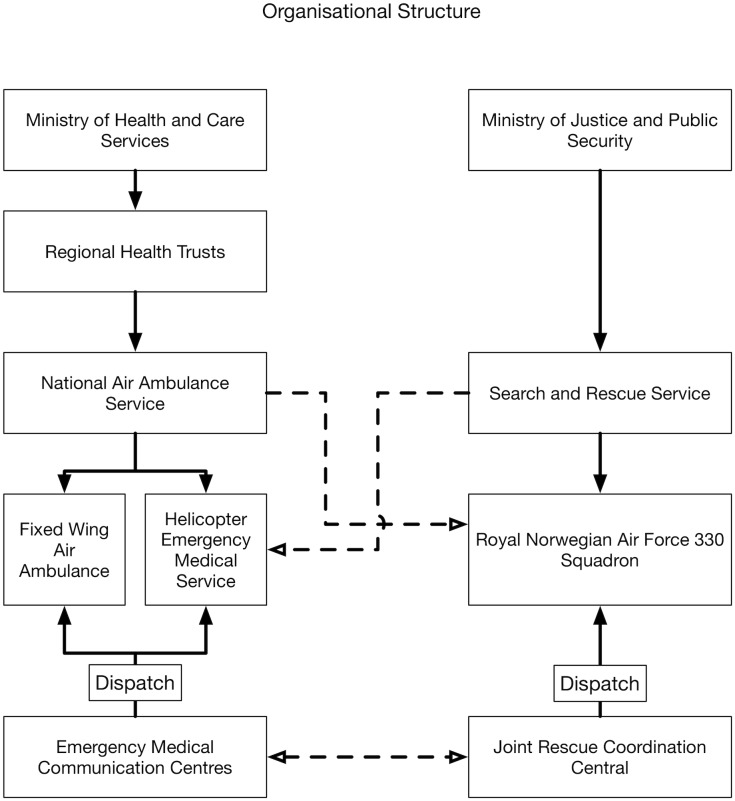
Organisational structure of Norwegian HEMS and SAR.

Norway has a national governmentally funded air ambulance service consisting of three elements; fixed-wing air ambulance, HEMS and SAR helicopters (Fig 1). The HEMS and fixed-wing air ambulance are the responsibility of the Ministry of Health and Care Services and are provided by the four government-owned regional health enterprises. The flight operation is contracted to commercial companies that operate on a strictly regulated contract and as an integral part of the national health care system. The SAR helicopters are the responsibility of the Ministry of Justice and Public Security. They are operated by the Royal Norwegian Air Force, but are per se a civilian resource and not subject to a military command structure in SAR or HEMS operations. The HEMS units are dispatched by the local medical communications centre (EMCC) responsible for the region where the HEMS is situated, while the SAR units are dispatched by one of two joint rescue coordination centres (JRCC). The SAR units are primarily used for SAR missions, but can be released for air ambulance missions by the JRCC on request from an EMCC and are therefore regarded as an integral part of the national air ambulance system. Similarly, HEMS can be released for SAR missions by the EMCC on request from the regional JRCC. Depending on the nature of the mission, EMCC or JRCC will have the main responsibility for coordinating resources. Medical staffing is similar in both HEMS and SAR with an anaesthesiologist and a rescue paramedic, but HEMS is only equipped for light SAR missions.

By the means of a cross-sectional survey we aimed to describe experiences with major incident management, preparedness and training among all Norwegian HEMS and SAR crewmembers to identify areas of improvement for major incident response and training programmes.

## Methods

### Study population

Norwegian HEMS crew configuration encompasses one pilot, one rescue paramedic and one consultant anaesthesiologist. This is the normal crew configuration, however at one HEMS base, a flight nurse supplements the crew. HEMS pilots are involved in on-scene medical care as long as it does not interfere with flight operations. All SAR units are staffed with two pilots, one flight-technician, one navigator, one rescue paramedic and one consultant anaesthesiologist. The national air ambulance service consisted of seven fixed-wing bases, 11 HEMS bases and seven SAR bases at the time of the study. All HEMS and SAR bases are equipped with a rapid response car for missions in the proximity of the base, or as a backup when weather or technical issues do not allow for the use of helicopters. Pilots, rescue paramedics and doctors working at HEMS and SAR bases as of January 1st 2015 were invited to participate in the study. Fixed-wing operations were excluded.

### Study design

A major incident was defined as an incident reported to EMCC or JRCC from pre-hospital resources as extensive enough to require extra personnel or resources from neighbouring districts and the activation of the emergency plans in involved hospitals. The magnitude of what constitutes a MI would vary according to resources available in the regions. This definition was included in the beginning of the survey to ensure that respondents understood what constituted a major incident. SAR helicopters are embedded in the air ambulance service and were defined as HEMS units.

We conducted a web-based (SurveyXact, (c) 2013–2015 Rambøll Management Consulting, Denmark) cross-sectional survey. Data was de-identified and collected in the period of the beginning of January 2015 to the end of June 2015. Eligible participants were invited individually via an e-mail describing the study. Non-responders received two reminders before they were excluded from the study. The program allowed only one answer per respondent and only sent reminders to non-responders.

In the absence of a validated questionnaire, our questions were constructed after inter-disciplinary consensus between HEMS pilot, rescue paramedic, doctor and researchers. Follow-up questions were designed to explore responder experiences in detail and to collapse irrelevant sections to avoid response fatigue. Some questions were profession specific (e.g. only for doctors), thereby changing the response nominator and denominator throughout the study.

The survey included three sections with questions pertaining to basic demographic data, experience from real incidents and training and equipment. The respondents were asked to relate questions regarding MI experiences to the latest MI they had attended within the last five years. If they had not attended any MIs within that period they only answered the training and equipment section.

Data were analyzed within SurveyXact and described by counts, median and inter quartile range (IQR). Being an anonymous survey, written consent to participate was not obtained. Responders agreed to participate in the study by answering the questionnaire. A disclaimer on personal privacy and ethical approval was presented to all potential responders in the first email that also described the authors and funding from Norwegian Air Ambulance Foundation. SurveyXact sent two reminders to non-responders before they were excluded from the study. Data was aggregated before analysis to avoid recognition of individual answers. The Regional Committee for Ethics in Medical Research concluded that ethical approval was not needed (2014/720/REK sør-øst D) and the Norwegian Social Science Data Services approved the study (38408).

## Results

### Study population

**Fig 2:**
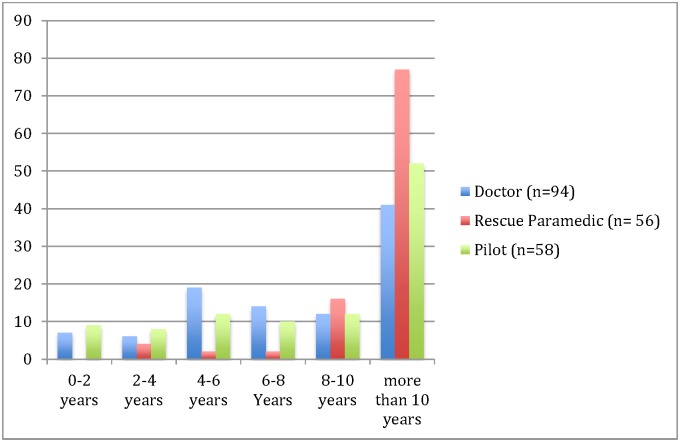
Years of experience working in HEMS/SAR and % of total respondents.

A total of 329 crewmembers were invited to participate in the survey and 229 (70%) responded. Rescue paramedics had the highest response rate (82%) followed by doctors (67%) and pilots (63%). Most respondents had more than 10 years HEMS or SAR experience (Fig 2).

The doctors had experience from a median of 1 (n = 101, IQR 0–5) MI, whereas rescue paramedics and pilots had experience from a median of 3 (n = 64, IQR 0–8 or more) and 2 (n = 64, IQR 0–6) MIs respectively. Further, more than half of the respondents (n = 52, (51%) doctors, n = 38, (59%) rescue paramedics and n = 38, (59%) pilots) had attended a MI within the last 5 years.

### Incident description

Road traffic incident was the most common cause of incident (n = 61, 48%). Rural area (n = 80, 63%) was the most frequent location and summer (n = 50, 40%) the busiest season.

Blunt trauma was the dominating type of injury in 59% (n = 51) followed by penetrating trauma (n = 14, 16%), hypothermia (n = 14, 16%) and burns (n = 13, 15%) as other frequent injuries. Further incidents descriptors are found in Table 1.

**Table 1:** Description of the last incident attended by the responders.

**Incident characteristics**	RTI	61 (48%)
	Fire	31 (24%)
	On going violence	26(20%)
	Bus	21 (17%)
	Avalanche	21 (17%)
	Industrial accident	19 (15%)
	Tunnel	16(13%)
	Boat	16(13%)
	Airplane/Helicopter	13 (10%)
	Large crowd	11 (9%)
	Train	9 (7%)
	Explosives	9 (7%)
	Weather-related	7 (6%)
	Dangerous goods	3 (2%)
	Tram	2 (2%)
	CBRNe	1 (1%)
**Location**	Rural	80 (63%)
	Mixed	37 (29%)
	Alpine	25 (20%)
	Urban	24 (19%)
	Maritime	17 (13%)
**Environment**	Daylight	90 (71%)
	Darkness	53 (42%)
	Rain	29 (23%)
	Fog	26 (21%)
	Snow	22 (17%)
	Storm	20 (16%)
**Season**	Summer	50 (40%)
	Winter	36 (29%)
	Autumn	31 (25%)
	Spring	8 (6%)

Note: n = 126, multiple answers allowed

RTI = Road traffic incidents, CBRNe = Chemical, Biological, Radioactive, Nuclear and Explosive

### Resources on-scene

The main tasks performed by the HEMS and SAR crews were patient treatment (n = 94, 76%), triage (n = 61, 49%) and transport to local hospital (n = 46, 37%) or directly to a trauma centre (n = 37, 30%). Overview over participating agencies and individual tasks of personnel are depicted in Tables 2 and 3.

**Table 2:** Participating agencies in major incident management.

Police	118 (95%)
Ambulance	116 (94%)
Fire	110 (89%)
Other HEMS/SAR	95 (77%)
Rapid response car with GP	53 (43%)
Non-governmental organizations	49 (40%)
Military	42 (34%)
Rapid response car with anaesthesiologist	38 (31%)
Civil protection agencies	33 (27%)

Note: n = 125, multiple answers allowed. GP = General practitioner

**Table 3:** HEMS/SAR tasks.

**Doctor**	Treatment	42 (84%)
	Transport	29 (58%)
	Triage	25 (50%)
	Medical incident commander	23 (46%)
	Other leadership tasks	8 (16%)
**Pilot**	Transport	26 (70%)
	Coordination of other HEMS units	19 (51%)
	Organizing landing site	12 (32%)
	SAR	9 (24%)
	Secure scene	5 (14%)
**Rescue paramedic**	Treatment	34 (92%)
	Transport	18 (51%)
	Triage	12 (34%)
	Securing scene	8 (23%)
	Ambulance Incident Commander	4(11%)
	Casualty clearing officer	2 (6%)

Note: Doctors: n = 50, Pilots: n = 37, Rescue Paramedic: n = 35. Multiple answers allowed.

In 32% (n = 40) of the incidents, HEMS and SAR transported extra personnel and extra equipment to scene in 52% (n = 64) of the incidents.

### Coordination and cooperation

The coordination and cooperation of multiple HEMS/SAR units on-scene are shown in Table 4.

**Table 4:** Coordination and cooperation.

**How many EMCCs did you contact from start to end of mission? Median (IQR)**		**1 (1–2)**
**Several HEMS/SAR units on-scene?**	Yes	98 (83%)
	No	16 (14%)
	Do not know	4 (3%)
**Who informed you of the other units?**	EMCC	27 (47%)
	JRCC	16 (28%)
	Other HEMS/SAR units	11 (19%)
	No information	3 (5%)
	Do not know	1 (2%)
**Who coordinated HEMS units on scene**	Own aircraft	34(59%)
	Other HEMS/SAR units	7 (12%)
	EMCC	5 (9%)
	Other	5 (9%)
	JRCC	2 (3%)
	Do not know	5 (9%)
**What type of communication was used to communicate with other units**	VHF	81 (45%)
**(more than one option possible)**	Norwegian public safety radio	53 (29%)
	Mobile phone	33 (18%)
	Annet	14 (7%)

Note: n = 118; IQR = Inter Quartile Range, EMCC = emergency medical communications centre, JRCC = joint rescue coordination centre, VHF = very high frequency

Guidelines for coordination of multiple units were available for 41% (n = 24) of the pilots. Among SAR pilots, 80% (n = 20) reported they lacked enough equipment for situational awareness, compared to 9% (n = 3) among the HEMS pilots.

Table 5 depict crew rating of key aspects of major incident management.

**Table 5:** Crew rating of selected key aspects of major incident management.

How would you rate	Median	IQR
On-scene management	4	(3–4)
Inter-disciplinary cooperation	4	(3–4)
Scene-safety	4	(4–5)
Personnel identification (tabards)	4	(3–4)
Personal protective equipment	4	(4–5)
Communication aids	2	(2–4)
Triage	4	(3–4)
Medical equipment	4	(4–5)

Note: n = 118, Rated on Likert scale 1–5. (1 = Very bad, 5 = Very good); IQR = Inter Quartile Range

### Equipment and training

Equipment available for major incident management include extra communication aids (n = 79, 38%), extra rescue technical kit (n = 156, 75%), triage tags (n = 177, 85%), stretchers (n = 204, 98%), anti-hypothermia kits (n = 175, 84%) and extra medical equipment (n = 166, 80%). When reporting on missing equipment, 38% (n = 75) reported communication aids, 16% (n = 31) extra medical equipment whereas 46% (n = 90) reported that they did not lack any extra equipment. Training for major incident management is depicted in Table 6.

**Table 6:** Training for major incident management and extra equipment available for handling major incidents (n = 209).

**How many times / year do you train for Major Incident management? Median (IQR)**	1 (0–2)
**How often do you train with other emergency services?**	
Always	44 (21%)
Sometimes	148 (71%)
Never	10 (5%)
Do not know	6 (3%)
**If yes, with who (n = 197, multiple answers allowed)**	
EMS	177 (90%)
Police	169 (86%)
Fire service	169 (86%)
NGO	143 (73%)
Other HEMS/SAR units	86 (44%)
Military	67 (34%)
Primary health care	60 (30%)
**What do you want more training for in the future: (n = 208, multiple answers allowed)**	
Management	75 (36%)
Communication	66 (32%)
Coordination	65 (31%)
Leadership	60 (29%)
Decision-making	50 (24%)
Triage	39 (19%)
Rescue technical procedures	22 (11%)
Medical procedures / knowledge	21 (10%)

Note: IQR = Inter Quartile Range; EMS = Emergency Medical Services; NGO = Non-Governmental Organization

## Discussion

This national cross-sectional survey found that approximately half of Norwegian HEMS and SAR crewmembers attended a MI during the last five years. Rescue paramedics and pilots had attended more MIs than doctors. The contribution of HEMS in MI management was typically patient treatment, triage and transport of patients and personnel. This echoes the findings from a recent systematic review on the use of HEMS in major incident management. [7] Interdisciplinary training on frequent scenarios with focus on cooperation and communication was called for by most respondents.

### Incident description and resources on-scene

Road traffic incidents were reported to be the most frequent cause of MI. More than half of the incidents took place in autumn and winter when daylight is limited. A recent study of Norwegian HEMS found that cancellations were more frequent at night-time and during autumn and winter. [8] Sub-arctic weather conditions and seasonal darkness makes flight conditions in Norway challenging. Requirements for visibility and cloud base are strict for HEMS missions during darkness and thereby causes more cancelling of missions when light is low. The Norwegian All weather SAR project aims to improve the bad weather capacity for the next generation SAR helicopters. [9] In addition the Norwegian Air Ambulance”Points IN Space” project with pre-fixed routes and the Norwegian Air Ambulance”Weather Camera project” aims to improve the regularity of the HEMS missions during darkness and austere weather. [10]

Most operations were conducted in rural areas, which coincide well with Norway being a sparsely populated country with vast distances and a sub-arctic climate. [11,12]

### Coordination and communication

The majority of incidents mobilised an interdisciplinary response. Close cooperation across hierarchical levels and knowledge of the skills of professionals and participating agencies is important in an emergency response with limited resources. [13–15] Critical decisions are made in early stages of the emergency response when resources are not meeting the demand and are made under time pressure. [16]

The HEMS and SAR crewmembers considered communication a challenge, echoing previous descriptions of overloaded networks. [13] In the 2011, Utøya incident communication was done on both the new Terrestrial Trunked Radio (TETRA) system and on the old analogue system, thereby complicating communication. [17] The pilots reported that they had to contact a median of two different ERCCs, but only in half of the incidents did ERCC inform of other aircraft involved thereby potentially increasing the risk for adverse aviation events. This indicates insufficient coordination procedures among ERCCs regarding resources involved. At the time of the survey, 11 ERCCs were involved in dispatching 11 HEMS. Fewer and larger ERCCs or fewer ERCCs involved in dispatching neighbouring HEMS units might be a solution. The pilots reported communication with other aircraft prior to arrival as bad, but good to very good on-scene. This might reflect limitations in the radio transmission range, but it may also reflect insufficient coordinating procedures by the ERCCs on a MI with multiple HEMS/SAR helicopters. The TETRA system was fully implemented in 2016 hopefully contributing to more secure and efficient communication. [18] Rapid access to essential information reduces risk during MIs. [19] Among the SAR pilots, 80% reported a lack of equipment for situational awareness while only 9% of the HEMS pilots answered that they lacked equipment for situational awareness. This discrepancy indicates clearly an improvement potential regarding the equipment on the SAR helicopters. The acquisition of the new all weather SAR helicopters may improve equipment status considerably. [9]

### Equipment and training

The extra equipment HEMS brought to scene was considered sufficient by 46% of the respondents, whereas 38% wanted more communication equipment. Although the crews train regularly, they call for more inter-disciplinary exercises that should focus on coordination, communication and cooperation. This study emphasise the importance of training on prevalent scenarios, such as road traffic incidents and austere weather conditions.

### Strengths and limitations

This study aimed to depict the inter-disciplinary cooperation by including all HEMS/SAR doctors, rescue paramedics and pilots in Norway. The study achieved a response rate of 70%, which is considered acceptable. [20] Although the study population only constituted 329 potential respondents, it depicts the entire Norwegian HEMS crewmember cohort. Approximately half of the respondents had attended a major incident the last five years. The lack of a uniformly accepted definition of a major incident remains a challenge. [4,21] The present definition was constructed to increase understanding of what constitutes a major incident from the respondents. Cross-sectional study design only depicts present state of major incident preparedness and experience; causal correlations cannot be made. We also cannot exclude a certain recall bias since some of the experiences reported took place up until five years ago. We think however that the potential risk of recall bias is outweighed by the number of incidents and amount of survey data this five-year period includes. A degree of selection bias can also not be excluded. Potential respondents who have never experienced a major incident may have neglected the survey causing a skewness in the material. We hope however that the relatively high response rate of 70% makes the results representative. Few studies on major incident management in Norway have been made and no validated questionnaire existed. The present questionnaire was designed after inter-disciplinary consensus.

## Conclusion

Norwegian HEMS and SAR crewmembers attend major incidents infrequently. Road traffic incidents constitute the majority of incidents and most operations are conducted in rural areas with blunt trauma as the dominating injury. HEMS predominately contribute with treatment, triage and transport of patients, equipment and personnel. Failing communication and inadequate air traffic control remains a challenge in the immediate inter-disciplinary response phase. More training with focus on coordination, communication and cooperation is called for.

## Supporting information

S1 File Survey. Original (Norwegian) version.
(DOC)

S2 File Survey. English version.
(DOC)

S3 File Data in Excel. Original (Norwegian) version.
(XLSX)

S4 File Data in Excel. English (translated) version.
(XLSX)

## Abbreviations

CBRNe: Chemical, Biological, Radioactive, Nuclear and Explosive
EMCC: emergency medical communications centre
EMS: emergency medical services
GP: General practitioner
HEMS: helicopter emergency medical services
IQR: inter quartile range
JRCC: joint rescue coordination centre
MI: major incident
NGO: Non-Governmental Organization
RTI: Road traffic incident
SAR: search and rescue
TETRA: terrestrial trunked radio
VHF: very high frequency
