# Supplementary material for: Helicopter emergency medical services in major incident management: A national Norwegian cross-sectional survey
Source: PLoS One. 2017 Feb 13;12(2):e0171436. doi: 10.1371/journal.pone.0171436 (PMC5305240; doi:10.1371/journal.pone.0171436)
Supplement: S1 File — Original (Norwegian) version. (DOC) [file pone.0171436.s001.doc]

| Til deg som jobber i luftambulansetjenesten / redningstjenesten i Norge. Tusen takk for at du tar deg tid til å besvare undersøkelsen.  Stor hendelse: En hendelse/ulykke som er så omfattende ut i fra melding til AMK at stedlige ressurser (ambulanse, politi og brann) må tilkalle mannskaper på ekstravakt eller hente inn ressurser fra nabodistrikt. Den er så stor at det slås katastrofealarm på nærmeste sykehus. Hendelsens størrelse og hva man kan håndtere vil variere ut i fra de ressurser man har til rådighet i sin region. |
| --- |

Hva er ditt yrke?

(1) ❑ Lege

(2) ❑ Redningsmann

(3) ❑ Pilot

I hvor mange store hendelser har du som innsatspersonell vært innvolvert i redningsarbeid?

(1) ❑ 0

(2) ❑ 1

(3) ❑ 2

(4) ❑ 3

(5) ❑ 4

(6) ❑ 5

(7) ❑ 6

(8) ❑ 7

(9) ❑ 8

(10) ❑ 9

(11) ❑ 10 eller flere

Har du vært innvolvert som innsatspersonell i en stor hendelse siste 5 år?

(1) ❑ Ja

(2) ❑ Nei

| Generelle kjennetegn ved hendelsen. Svar på de neste spørsmålene ut i fra den siste store hendelsen du var på. |
| --- |

Hvilken tjeneste jobbet du i ved den aktuelle hendelsen?

(1) ❑ SAR / redningshelikoptertjenesten

(2) ❑ Rotorwing luftambulansetjenesten

Beskriv hendelsen (sett gjerne flere kryss).

(1) ❑ Stor trafikkulykke

(2) ❑ Buss

(3) ❑ Tog

(4) ❑ Trikk

(5) ❑ Fly / Helikopter

(6) ❑ Tunnel

(7) ❑ Båt

(8) ❑ Ekstremvær

(9) ❑ Skred

(10) ❑ Arbeidsulykke / Industriulykke

(12) ❑ Brann

(13) ❑ Stor folkemengde

(14) ❑ Eksplosiver

(15) ❑ Skarp situasjon / skyting pågår

(16) ❑ Kjemisk / Biologisk / Radioaktiv / Nukleær

(17) ❑ Farlig gods

(18) ❑ Ukjent

(19) ❑ Annet _____

Hvor var hendelsen? (Sett gjerne flere kryss)

(1) ❑ By

(2) ❑ Tettbygd strøk

(3) ❑ Grisgrendt strøk

(4) ❑ Maritim

(5) ❑ Fjell

(6) ❑ Annet _____

Værforhold ved hendelsens start. (Sett gjerne flere kryss)

(1) ❑ Dagslys

(2) ❑ Mørke

(3) ❑ Snøvær

(4) ❑ Tåke

(5) ❑ Regn

(6) ❑ Sterk vind / storm

(7) ❑ Annet _____

Årstid.

(1) ❑ Sommer

(2) ❑ Høst

(3) ❑ Vinter

(4) ❑ Vår

Hvilke pasientskader dominerte? (Sett gjerne flere kryss)

(1) ❑ Stumpe skader

(2) ❑ Spisse skader

(3) ❑ Hypotermi

(4) ❑ Brannskader

(5) ❑ Andre __________

Hvilke andre etater deltok i redningsarbeidet? (Sett gjerne flere kryss)

(1) ❑ Ambulanse

(2) ❑ Legebil med legevaktslege

(3) ❑ Legebil med anestesilege

(5) ❑ Brannvesen

(6) ❑ Politi

(7) ❑ Andre luftambulansetjenester / redningshelikoptre

(8) ❑ Frivillige organisasjoner

(9) ❑ Sivilforsvaret

(13) ❑ Forsvaret

(10) ❑ Industrivern

(11) ❑ Utenlandske enheter

(12) ❑ Andre _____

| Arbeid på skadested. |
| --- |

Hva ble *ditt crew og fartøy* brukt til? (sett gjerne flere krys*s)*

(1) ❑ Transport til skadested med ekstra ressurser / innsatspersonell

(2) ❑ Sikring av skadested

(3) ❑ Ledelsesoppgaver / koordinering

(4) ❑ Triage

(5) ❑ Pasientbehandling

(6) ❑ Transport fra skadested til samleplass

(7) ❑ Transport fra skadested til akuttsykehus

(8) ❑ Transport fra skadested til regionalt traumesenter

(9) ❑ Transport fra samleplass til akuttsykehus

(10) ❑ Transport fra samleplass til regionalt traumesenter

(11) ❑ Transport fra akuttsykehus til regionalt traumesenter

(12) ❑ Søk

(13) ❑ Annet _____

Hva ble *du* brukt til? (Sett gjerne flere kryss)

(1) ❑ Fagleder helse

(2) ❑ Annen ledelse

(3) ❑ Triage

(4) ❑ Pasientbehandling

(5) ❑ Transport

(6) ❑ Annet __________

Hva ble *du* brukt til? (Sett gjerne flere kryss)

(1) ❑ Sikring av skadested

(2) ❑ Ledelsesoppgaver

(3) ❑ Koordinering av andre luftfartøy

(9) ❑ Organisering / tilrettelegging av landingsplass

(4) ❑ Triage

(5) ❑ Behandling

(6) ❑ Transport

(7) ❑ Søk

(8) ❑ Annet __________

Hva ble *du* brukt til? (Sett gjerne flere kryss)

(1) ❑ Redningsmann(assistent til legen)

(2) ❑ Operativ leder helse

(3) ❑ Leder samleplass

(4) ❑ Leder skadested

(5) ❑ Leder innbringertjeneste

(6) ❑ Triage

(7) ❑ Pasientbehandling

(8) ❑ Transport

(9) ❑ Sikring av skadested

(10) ❑ Søk

(11) ❑ Annet __________

Tok dere med ekstra personell utover vanlig crew?

(1) ❑ Ja

(2) ❑ Nei

Hvilke yrkeskategorier? (Sett gjerne flere kryss)

(1) ❑ Lege

(2) ❑ Sykepleier

(3) ❑ Redningsmann

(4) ❑ Pilot

(5) ❑ Ambulansepersonell

(6) ❑ Hospitant

(7) ❑ Andre __________

Tok dere med ekstra utstyr?

(1) ❑ Ja

(2) ❑ Nei

(3) ❑ Vet ikke

Hvilket utstyr tok dere med? (Sett gjerne flere kryss)

(1) ❑ Sambandsutstyr

(2) ❑ Redningsteknisk utstyr

(3) ❑ Triageutstyr

(4) ❑ Bårer

(5) ❑ Medikamenter

(6) ❑ Hypotermiforebyggende utstyr

(7) ❑ Medisinsk ekstrautstyr

(8) ❑ Annet __________

Hadde dere tilstrekkelig utstyr for å håndtere situasjonen?

(1) ❑ Ja

(2) ❑ Nei

Hvilket utstyr manglet? (Sett gjerne flere kryss)

(1) ❑ Sambandsutstyr

(2) ❑ Redningsteknisk utstyr

(3) ❑ Triageutstyr

(4) ❑ Bårer

(5) ❑ Medikamenter

(6) ❑ Hypotermiforebyggende utstyr

(7) ❑ Medisinsk ekstrautstyr

(8) ❑ Annet __________

Ble pasienter triagert på en systematisk måte?

(1) ❑ Ja

(2) ❑ Nei

(3) ❑ Vet ikke

Hvilken type triage ble brukt?

(1) ❑ TAS triage

(2) ❑ SALT

(3) ❑ Nasjonal veileder for masseskadetriage

(4) ❑ Ingen formell triage ble benyttet

(5) ❑ Annen _____

Hvordan fungerte:

|  | Svært dårlig | Dårlig | Hverken dårlig eller bra | Bra | Svært bra | Vet ikke |
| --- | --- | --- | --- | --- | --- | --- |
| Skadestedsledelse | (1) ❑ | (2) ❑ | (3) ❑ | (4) ❑ | (5) ❑ | (6) ❑ |
| Felles forståelse for organisering av skadested | (1) ❑ | (2) ❑ | (3) ❑ | (4) ❑ | (5) ❑ | (6) ❑ |
| Egensikkerhet | (1) ❑ | (2) ❑ | (3) ❑ | (4) ❑ | (5) ❑ | (6) ❑ |
| Merking av personell | (1) ❑ | (2) ❑ | (3) ❑ | (4) ❑ | (5) ❑ | (6) ❑ |
| Personlig bekledning | (1) ❑ | (2) ❑ | (3) ❑ | (4) ❑ | (5) ❑ | (6) ❑ |
| Samband | (1) ❑ | (2) ❑ | (3) ❑ | (4) ❑ | (5) ❑ | (6) ❑ |
| Triage | (1) ❑ | (2) ❑ | (3) ❑ | (4) ❑ | (5) ❑ | (6) ❑ |
| Medisinsk utstyr | (1) ❑ | (2) ❑ | (3) ❑ | (4) ❑ | (5) ❑ | (6) ❑ |
| Ekstra båremateriell | (1) ❑ | (2) ❑ | (3) ❑ | (4) ❑ | (5) ❑ | (6) ❑ |
| Annet beredskapsutstyr | (1) ❑ | (2) ❑ | (3) ❑ | (4) ❑ | (5) ❑ | (6) ❑ |

| Sikkerhet, samarbeid og logistikk. |
| --- |

Hvor mange AMK sentraler var dere i kontakt med fra alarmering til oppdraget var gjennomført og dere var tilbake på basen?

(1) ❑ 0

(2) ❑ 1

(3) ❑ 2

(4) ❑ 3

(5) ❑ 4

(6) ❑ 5

(7) ❑ 6 eller flere

Ble det rekvirert flere helikoptre til hendelsen?

(1) ❑ Ja

(2) ❑ Nei

(3) ❑ Vet ikke

Hvor mange helikoptre var innvolvert?

__________

Hvem informerte om at disse var involvert / rekvirert? (Sett gjerne flere kryss)

(1) ❑ AMK

(2) ❑ HRS

(3) ❑ ATC

(4) ❑ Andre luftambulanser / redningshelikoptre

(5) ❑ Andre nødetater

(6) ❑ Fikk ingen informasjon

(7) ❑ Vet ikke

Hvem påtok seg den koordinerende rollen for samvirke med øvrige helikoptre på skadested?

(1) ❑ AMK

(2) ❑ HRS

(3) ❑ ATC

(4) ❑ Andre helikoptre

(5) ❑ Eget helikopter / fartøysjef

(6) ❑ Andre __________

(7) ❑ Vet ikke / ikke aktuelt

På hvilket samband foregikk kommunikasjon med øvrige helikoptre? (Sett gjerne flere kryss)

(1) ❑ Helseradio / digitalt nødnett

(2) ❑ VHF flyradio

(3) ❑ Mobiltelefon

(4) ❑ Annet __________

Hvordan vil du vurdere kommunikasjon med øvrige helikoptre før ankomst skadested?

(1) ❑ Svært dårlig

(2) ❑ Dårlig

(3) ❑ Hverken dårlig eller god

(4) ❑ God

(5) ❑ Svært god

(6) ❑ Vet ikke / ikke aktuelt

Hvordan vil du vurdere *samarbeid og kommunikasjon* under innsatsen med:

|  | Svært dårlig | Dårlig | Hverken eller | Bra | Svært bra | Vet ikke / Ikke aktuelt |
| --- | --- | --- | --- | --- | --- | --- |
| Piloter | (1) ❑ | (2) ❑ | (3) ❑ | (4) ❑ | (5) ❑ | (6) ❑ |
| Redningsmenn | (1) ❑ | (2) ❑ | (3) ❑ | (4) ❑ | (5) ❑ | (6) ❑ |
| LAT leger | (1) ❑ | (2) ❑ | (3) ❑ | (4) ❑ | (5) ❑ | (6) ❑ |
| Andre leger | (1) ❑ | (2) ❑ | (3) ❑ | (4) ❑ | (5) ❑ | (6) ❑ |
| Eget crew | (1) ❑ | (2) ❑ | (3) ❑ | (4) ❑ | (5) ❑ | (6) ❑ |
| Ansvarlig AMK | (1) ❑ | (2) ❑ | (3) ❑ | (4) ❑ | (5) ❑ | (6) ❑ |
| Ansvarlig HRS | (1) ❑ | (2) ❑ | (3) ❑ | (4) ❑ | (5) ❑ | (6) ❑ |
| Ansvarlig ATC | (1) ❑ | (2) ❑ | (3) ❑ | (4) ❑ | (5) ❑ | (6) ❑ |
| Ansvarlig lokal redningssentral (LRS) | (1) ❑ | (2) ❑ | (3) ❑ | (4) ❑ | (5) ❑ | (6) ❑ |
| Ambulansetjenesten | (1) ❑ | (2) ❑ | (3) ❑ | (4) ❑ | (5) ❑ | (6) ❑ |
| Politi på skadested | (1) ❑ | (2) ❑ | (3) ❑ | (4) ❑ | (5) ❑ | (6) ❑ |
| Brannvesen | (1) ❑ | (2) ❑ | (3) ❑ | (4) ❑ | (5) ❑ | (6) ❑ |
| Forsvaret | (1) ❑ | (2) ❑ | (3) ❑ | (4) ❑ | (5) ❑ | (6) ❑ |
| Frivillige organisasjoner | (1) ❑ | (2) ❑ | (3) ❑ | (4) ❑ | (5) ❑ | (6) ❑ |
| Sivilforsvaret | (1) ❑ | (2) ❑ | (3) ❑ | (4) ❑ | (5) ❑ | (6) ❑ |
| Mottagende sykehus | (1) ❑ | (2) ❑ | (3) ❑ | (4) ❑ | (5) ❑ | (6) ❑ |
| Avleverende sykehus ved sekundærtransport | (1) ❑ | (2) ❑ | (3) ❑ | (4) ❑ | (5) ❑ | (6) ❑ |
| Industrivern | (1) ❑ | (2) ❑ | (3) ❑ | (4) ❑ | (5) ❑ | (6) ❑ |

Evt beskriv hva som var bra / utfordrende med samhandlingen.

________________________________________
________________________________________
________________________________________
________________________________________
________________________________________
________________________________________
________________________________________
________________________________________

I det aktuelle området for hendelsen, hvordan vil du vurdere din oversikt over:

|  | Svært dårlig | Dårlig | Hverken dårlig eller god | God | Svært god | Vet ikke / ikke aktuelt |
| --- | --- | --- | --- | --- | --- | --- |
| Sykehus | (1) ❑ | (2) ❑ | (3) ❑ | (4) ❑ | (5) ❑ | (6) ❑ |
| Legevakt | (1) ❑ | (2) ❑ | (3) ❑ | (4) ❑ | (5) ❑ | (6) ❑ |
| Ambulansetjeneste | (1) ❑ | (2) ❑ | (3) ❑ | (4) ❑ | (5) ❑ | (6) ❑ |

| Generelt vedr samarbeid / logistikk. |
| --- |

Hvem bør, etter din mening, koordinere lufttrafikken i ukontrollert luftrom med flere deltagende helikoptre ved en stor hendelse?

(1) ❑ AMK

(2) ❑ ATC

(3) ❑ HRS

(4) ❑ Første ambulanse/redningshelikopter på stedet

(5) ❑ Andre _____

(6) ❑ Vet ikke

Finnes det retningslinjer for hvordan koordinering / samvirke mellom flere helikoptre på skadested i ukontrollert luftrom skal foregå i ditt operatørselskap / skvadron?

(1) ❑ Ja

(2) ❑ Nei

(3) ❑ Vet ikke

Er helikopteret du opererer, etter din mening, utstyrt med tilstrekkelig med nødvendig teknisk utstyr for å gi "situational awareness" i forhold til flysikkerhet og andre helikoptre?

(1) ❑ Ja

(2) ❑ Nei

(3) ❑ Vet ikke

Hvilket utstyr savner du?

________________________________________________________________________________
________________________________________________________________________________
________________________________________________________________________________
________________________________________________________________________________
________________________________________________________________________________
________________________________________________________________________________
________________________________________________________________________________
________________________________________________________________________________
________________________________________________________________________________
________________________________________________________________________________
________________________________________________________________________________
________________________________________________________________________________
________________________________________________________________________________
________________________________________________________________________________
________________________________________________________________________________

Kjenner du til hvilken nasjonal VHF flyfrekvens som vanligvis benyttes under redningsoperasjoner?

(1) ❑ Ja

(2) ❑ Nei

Hvordan vil du vurdere din oversikt over:

|  | Svært dårlig | Dårlig | Hverken dårlig eller god | God | Svært god | Vet ikke |
| --- | --- | --- | --- | --- | --- | --- |
| Andre redningsressurser i din bases primærområde | (1) ❑ | (2) ❑ | (3) ❑ | (4) ❑ | (5) ❑ | (6) ❑ |
| Behandlingstilbud ved sykehus i din bases primærområde | (1) ❑ | (2) ❑ | (3) ❑ | (4) ❑ | (5) ❑ | (6) ❑ |
| Andre redningsressurser utenfor din bases primærområde | (1) ❑ | (2) ❑ | (3) ❑ | (4) ❑ | (5) ❑ | (6) ❑ |
| Sykehusstrukturen utenfor din bases primærområde | (1) ❑ | (2) ❑ | (3) ❑ | (4) ❑ | (5) ❑ | (6) ❑ |

| Kompetanse og prosedyrer. |
| --- |

Har du erfaring i å være Fagleder Helse?

(1) ❑ Ja

(2) ❑ Nei

Har du vært på en stor hendelse hvor du, etter din mening, burde ha vært Fagleder Helse?

(1) ❑ Ja

(2) ❑ Nei

Har du vært på en stor hendelse hvor du var Fagleder Helse, hvor dette var unødvendig?

(1) ❑ Ja

(2) ❑ Nei

Har du erfaring i å være Operativ Leder Helse?

(1) ❑ Ja

(2) ❑ Nei

Har du vært på en stor hendelse hvor du, etter din mening, burde ha vært Operativ Leder Helse?

(1) ❑ Ja

(2) ❑ Nei

Har du vært på en stor hendelse hvor du var Operativ Leder Helse, hvor dette var unødvendig?

(1) ❑ Ja

(2) ❑ Nei

Hvordan vurderer du:

|  | Svært dårlig | Dårlig | Hverken dårlig eller god | God | Svært god | Vet ikke |
| --- | --- | --- | --- | --- | --- | --- |
| Din egen kompetanse til å organisere et skadested i forbindelse med en stor hendelse? | (1) ❑ | (2) ❑ | (3) ❑ | (4) ❑ | (5) ❑ | (6) ❑ |
| Opplæringen du har fått i å organisere et skadested ved en stor hendelse? | (1) ❑ | (2) ❑ | (3) ❑ | (4) ❑ | (5) ❑ | (6) ❑ |
| Din egen kompetanse for å ivareta rollen som Fagleder Helse? | (1) ❑ | (2) ❑ | (3) ❑ | (4) ❑ | (5) ❑ | (6) ❑ |
| Opplæringen du har fått for å ivareta rollen som Fagleder Helse? | (1) ❑ | (2) ❑ | (3) ❑ | (4) ❑ | (5) ❑ | (6) ❑ |
| Din egen kompetanse for å ivareta rollen som Operativ Leder Helse? | (1) ❑ | (2) ❑ | (3) ❑ | (4) ❑ | (5) ❑ | (6) ❑ |
| Opplæringen du har fått for å ivareta rollen som Operativ Leder Helse? | (1) ❑ | (2) ❑ | (3) ❑ | (4) ❑ | (5) ❑ | (6) ❑ |

Har du gjennomført kurset "Samvirke på skadested"?

(1) ❑ Ja

(2) ❑ Nei

Hvor mange år er det siden du gjennomførte kurset?

(1) ❑ 0

(2) ❑ 1

(3) ❑ 2

(4) ❑ 3

(5) ❑ 4 eller mer

Hvordan vil du vurdere din utdanning i å håndtere dine arbeidsoppgaver i en stor hendelse?

(1) ❑ Svært dårlig / manglende

(2) ❑ Dårlig

(3) ❑ Hverken dårlig eller god

(4) ❑ God

(5) ❑ Svært god

Hvor mange ganger per år trener du på store hendelser? (Er du tilknyttet flere baser, svar på totalt antall trening)

(1) ❑ 0

(2) ❑ 1

(3) ❑ 2

(4) ❑ 3

(5) ❑ 4 eller flere

Hvor ofte trener *din tjeneste* med andre etater ved større øvelser?

(1) ❑ Hver gang

(2) ❑ Av og til

(3) ❑ Aldri

(4) ❑ Vet ikke

Med hvem?

(1) ❑ Politi

(2) ❑ Brann

(3) ❑ Ambulanse

(4) ❑ Andre luftambulanser / redningshelikoptre

(5) ❑ Primærhelsetjenesten (legevakt / kommunehelsetjenesten)

(9) ❑ Utrykningspersonell fra sykehus

(6) ❑ Forsvaret

(7) ❑ Frivillige organisasjoner

(8) ❑ Andre _____

På en skala fra 1 til 5 hvor 1 er liten og 5 er stor grad, vil mer kunnskap og øvelse gjøre deg bedre forberedt ved større hendelser i fremtiden?

(1) ❑ 1

(2) ❑ 2

(3) ❑ 3

(4) ❑ 4

(5) ❑ 5

Hva ønsker du mer kunnskap / øvelse på? (Sett gjerne mer enn ett kryss)

(1) ❑ Ledelse

(2) ❑ Beslutningsprosess

(3) ❑ Organisering

(4) ❑ Kommunikasjon

(5) ❑ Samhandling med andre og egne etater

(6) ❑ Redningstekniske prosedyrer

(7) ❑ Medisinske prosedyrer / kunnskap

(8) ❑ Triage

(9) ❑ Annet _____

(10) ❑ Ingenting

Har du fått opplæring i den nye veilederen for masseskadetriage?

(1) ❑ Ja

(2) ❑ Nei

(3) ❑ Vet ikke

Har din tjeneste merkeutstyr som er tilpasset den nye veilederen for masseskadetriage?

(1) ❑ Ja

(2) ❑ Nei

(3) ❑ Vet ikke

Hva finnes av ekstrautstyr til bruk ved store hendelser i din tjeneste? (Sett gjerne mer enn ett kryss)

(1) ❑ Sambandsutstyr

(2) ❑ Redningsteknisk utstyr

(3) ❑ Triageutstyr

(4) ❑ Bårer

(6) ❑ Hypotermiforebyggende utstyr

(7) ❑ Medisinsk ekstrautstyr

(8) ❑ Annet _____

(9) ❑ Ingenting

Hva savner du av ekstrautstyr til bruk ved store hendelser i din tjeneste?

(1) ❑ Sambandsutstyr

(2) ❑ Redningsteknisk utstyr

(3) ❑ Triageutstyr

(4) ❑ Bårer

(5) ❑ Hypotermiforebyggende utstyr

(6) ❑ Medisinsk ekstrautstyr

(7) ❑ Annet _____

(8) ❑ Ingenting

| Til slutt litt generelt. |
| --- |

Hvor mange år har du jobbet innen pre-hospitale tjenester?

(1) ❑ 0-2

(2) ❑ 2-4

(3) ❑ 4-6

(4) ❑ 6-8

(5) ❑ 8-10

(6) ❑ mer enn 10 år

Hvor jobber du?

(1) ❑ SAR / redningshelikoptertjenesten

(2) ❑ Rotorwing luftambulansetjenesten

Hvilken helseregion jobber du i?

(1) ❑ Nord

(2) ❑ Midt

(3) ❑ Vest

(4) ❑ Sør-Øst

Har du noen innspill til håndtering av store hendelser? Forbedringspotensialer eller hva som fungerer bra / dårlig?

____________________________________________________________
____________________________________________________________
____________________________________________________________
____________________________________________________________
____________________________________________________________
____________________________________________________________
____________________________________________________________
____________________________________________________________
____________________________________________________________
____________________________________________________________
____________________________________________________________
____________________________________________________________

| *Tusen takk for at du tok deg tid til undersøkelsen.* |
| --- |
