# Supplementary material for: Helicopter emergency medical services in major incident management: A national Norwegian cross-sectional survey
Source: PLoS One. 2017 Feb 13;12(2):e0171436. doi: 10.1371/journal.pone.0171436 (PMC5305240; doi:10.1371/journal.pone.0171436)
Supplement: S2 File — English version. (DOC) [file pone.0171436.s002.doc]

| To those working in Helicopter Emergency Medical Services (HEMS) and Search and Rescue (SAR) services in Norway. We appreciate that you take the time to respond to the present survey.    **Definition:** **Major incident:** An incident reported to Emergency Medical Communication Centrals (EMCC) or Joint Rescue Coordination Centres (JRCC) from pre-hospital resources as extensive enough to require extra personnel or resources from neighbouring districts and the activation of the emergency plans in involved hospitals.  The magnitude of what constitutes a major incident will vary according to resources available in the regions.  **Survey:** |
| --- |

What is your profession?

(1) ❑ Doctor

(2) ❑ Rescue paramedic

(3) ❑ Pilot

In how many major incidents have you been involved as rescue personnel?

(1) ❑ 0

(2) ❑ 1

(3) ❑ 2

(4) ❑ 3

(5) ❑ 4

(6) ❑ 5

(7) ❑ 6

(8) ❑ 7

(9) ❑ 8

(10) ❑ 9

(11) ❑ 10 or more

Have you been involved in the management of a major incident the last 5 years?

(1) ❑ Yes

(2) ❑ No

| **Characteristics with the major incident:** -Please reply to the following questions based on the last major incident you attended. |
| --- |

**In which service did you work?**

(1) ❑ SAR/Recue helicopter service

(2) ❑ HEMS

Describe the incident: (Multiple alternatives allowed)

(1) ❑ Large road traffic incident

(2) ❑ Bus

(3) ❑ Train

(4) ❑ Tram/Underground

(5) ❑ Plane/Helicopter

(6) ❑ Tunnel

(7) ❑ Boat

(8) ❑ Extreme weather

(9) ❑ Avalanche

(10) ❑ Work related incident/Industrial incident

(12) ❑ Fire

(13) ❑ Large crowd

(14) ❑ Explosives

(15) ❑ Ongoing life-threatening violence - Active shooter

(16) ❑ Chemical/Biological/Radioactive/Nuclear

(17) ❑ Dangerous goods

(18) ❑ Unknown

(19) ❑ Other _____

**Where was the incident?** (Multiple alternatives allowed)

(1) ❑ City

(2) ❑ Urban area

(3) ❑ Rural area

(4) ❑ Maritime

(5) ❑ Alpine

(6) ❑ Other _____

Weather / conditions at incident start: (Multiple alternatives allowed)

(1) ❑ Daylight

(2) ❑ Darkness

(3) ❑ Snow

(4) ❑ Fog

(5) ❑ Rain

(6) ❑ Strong winds/storm

(7) ❑ Other _____

Season:

(1) ❑ Summer

(2) ❑ Autumn

(3) ❑ Winter

(4) ❑ Spring

Dominating type of injury? (Multiple alternatives allowed)

(1) ❑ Blunt

(2) ❑ Penetrating

(3) ❑ Hypothermia

(4) ❑ Burns

(5) ❑ Other __________

Which other services participated in the rescue work? (Multiple alternatives allowed)

(1) ❑ Ambulance

(2) ❑ Rapid response car with General Practitioner on-call

(3) ❑ Rapid response car with Anaesthesiologist

(5) ❑ Fire services

(6) ❑ Police

(7) ❑ Other HEMS/SAR services

(8) ❑ Voluntary organizations

(9) ❑ Civil defense

(13) ❑ Defense

(10) ❑ Industry protection services

(11) ❑ Foreign services

(12) ❑ Other _____

| **On-scene management:** |
| --- |

**What was your crew and aircraft used for?** (Multiple alternatives allowed)

(1) ❑ Transport to scene with extra resources/rescue personnel

(2) ❑ Securing scene

(3) ❑ Leadership/Coordination

(4) ❑ Triage

(5) ❑ Patient treatment

(6) ❑ Transport from scene to casualty clearing station

(7) ❑ Transport from scene to trauma unit

(8) ❑ Transport from scene to regional trauma center

(9) ❑ Transport from casualty clearing station to trauma unit

(10) ❑ Transport from casualty clearing station to regional trauma center

(11) ❑ Transport from trauma unit to regional trauma center

(12) ❑ Search

(13) ❑ Other _____

What was your role on-scene? (Multiple alternatives allowed)

(1) ❑ Medical incident officer (Fagleder helse)

(2) ❑ Other leadership

(3) ❑ Triage

(4) ❑ Patient treatment

(5) ❑ Transport

(6) ❑ Other __________

What was your duties on-scene? (Multiple alternatives allowed)

(1) ❑ Securing scene

(2) ❑ Leadership

(3) ❑ Coordination of other aircrafts

(9) ❑ Organization/Preparation of landing site

(4) ❑ Triage

(5) ❑ Treatment

(6) ❑ Transport

(7) ❑ Search

(8) ❑ Other __________

What was your duties on-scene? (Multiple alternatives allowed)

(1) ❑ Rescue paramedic (assisting the Doctor)

(2) ❑ Medical on-scene commander (Operativ leder helse)

(3) ❑ Leader casualty clearing station

(4) ❑ Leader incident scene

(5) ❑ Leader patient transport

(6) ❑ Triage

(7) ❑ Patient treatment

(8) ❑ Transport

(9) ❑ Securing scene

(10) ❑ Search

(11) ❑ Other __________

**Did you bring extra personnel in addition to ordinary crew?**

(1) ❑ Yes

(2) ❑ No

What professional category? (Multiple alternatives allowed)

(1) ❑ Doctor

(2) ❑ Nurse

(3) ❑ Rescue paramedic

(4) ❑ Pilot

(5) ❑ Ambulance personnel

(6) ❑ Observer

(7) ❑ Other __________

Did you bring extra equipment?

(1) ❑ Yes

(2) ❑ No

(3) ❑ Unknown

What type of equipment did you bring? (Multiple alternatives allowed)

(1) ❑ Communication equipment

(2) ❑ Rescue technical equipment

(3) ❑ Triage equipment

(4) ❑ Stretchers

(5) ❑ Drugs

(6) ❑ Hypothermia preventive equipment

(7) ❑ Medical supply

(8) ❑ Other __________

Did you have sufficient equipment to handle the situation?

(1) ❑ Yes

(2) ❑ No

What equipment was missing? (Multiple alternatives allowed)

(1) ❑ Communication equipment

(2) ❑ Rescue technical equipment

(3) ❑ Triage equipment

(4) ❑ Stretchers

(5) ❑ Drugs

(6) ❑ Hypothermia preventive equipment

(7) ❑ Medical supply

(8) ❑ Other __________

Where the patients systematically triaged?

(1) ❑ Yes

(2) ❑ No

(3) ❑ Unknown

What system for triage was used?

(1) ❑ TAS triage

(2) ❑ SALT

(3) ❑ National guidelines for major incident triage

(4) ❑ No formal system for triage was used

(5) ❑ Other _____

How did this work?

|  | Very bad | Bad | Neither good nor bad | Good | Very good | Unknown |
| --- | --- | --- | --- | --- | --- | --- |
| Scene management | (1) ❑ | (2) ❑ | (3) ❑ | (4) ❑ | (5) ❑ | (6) ❑ |
| Common understanding of scene organization | (1) ❑ | (2) ❑ | (3) ❑ | (4) ❑ | (5) ❑ | (6) ❑ |
| Own safety | (1) ❑ | (2) ❑ | (3) ❑ | (4) ❑ | (5) ❑ | (6) ❑ |
| Personnel tabards | (1) ❑ | (2) ❑ | (3) ❑ | (4) ❑ | (5) ❑ | (6) ❑ |
| PPE | (1) ❑ | (2) ❑ | (3) ❑ | (4) ❑ | (5) ❑ | (6) ❑ |
| Communication | (1) ❑ | (2) ❑ | (3) ❑ | (4) ❑ | (5) ❑ | (6) ❑ |
| Triage | (1) ❑ | (2) ❑ | (3) ❑ | (4) ❑ | (5) ❑ | (6) ❑ |
| Medical supply | (1) ❑ | (2) ❑ | (3) ❑ | (4) ❑ | (5) ❑ | (6) ❑ |
| Extra stretchers | (1) ❑ | (2) ❑ | (3) ❑ | (4) ❑ | (5) ❑ | (6) ❑ |
| Other preparedness equipment | (1) ❑ | (2) ❑ | (3) ❑ | (4) ❑ | (5) ❑ | (6) ❑ |

| **Safety, cooperation and logistics:** |
| --- |

How many EMCCs did you communicate with from activation until mission was completed and you had returned back to base?

(1) ❑ 0

(2) ❑ 1

(3) ❑ 2

(4) ❑ 3

(5) ❑ 4

(6) ❑ 5

(7) ❑ 6 or more

Where multiple helicopters activated to the incident?

(1) ❑ Yes

(2) ❑ No

(3) ❑ Unknown

How many helicopters were involved?

__________

Who informed that these helicopters were activated? (Multiple alternatives allowed)

(1) ❑ EMCC

(2) ❑ JRCC

(3) ❑ ATC

(4) ❑ Other HEMS/SAR helicopters

(5) ❑ Other rescue services

(6) ❑ No information given

(7) ❑ Unknown

Who coordinated cooperation between different helicopters on-scene?

(1) ❑ EMCC

(2) ❑ JRCC

(3) ❑ ATC

(4) ❑ Other helicopters

(5) ❑ Own helicopter/Captain

(6) ❑ Other __________

(7) ❑ Unknown / Not applicable

What mode of communication was used between the different helicopters? (Multiple alternatives allowed)

(1) ❑ Health radio/Digital emergency radio

(2) ❑ VHF flight radio

(3) ❑ Mobile telephone

(4) ❑ Other __________

How did communication with other helicopters prior to arrival to scene work?

(1) ❑ Very bad

(2) ❑ Bad

(3) ❑ Neither bad nor good

(4) ❑ Good

(5) ❑ Very good

(6) ❑ Unknown / not applicable

How did on-scene *cooperation and communication* work with:

|  | Very bad | Bad | Neither good nor bad | Good | Very good | Unknown |
| --- | --- | --- | --- | --- | --- | --- |
| Pilots | (1) ❑ | (2) ❑ | (3) ❑ | (4) ❑ | (5) ❑ | (6) ❑ |
| Rescue paramedic | (1) ❑ | (2) ❑ | (3) ❑ | (4) ❑ | (5) ❑ | (6) ❑ |
| HEMS Doctors | (1) ❑ | (2) ❑ | (3) ❑ | (4) ❑ | (5) ❑ | (6) ❑ |
| Other Doctors | (1) ❑ | (2) ❑ | (3) ❑ | (4) ❑ | (5) ❑ | (6) ❑ |
| Own crew | (1) ❑ | (2) ❑ | (3) ❑ | (4) ❑ | (5) ❑ | (6) ❑ |
| EMCC in-charge | (1) ❑ | (2) ❑ | (3) ❑ | (4) ❑ | (5) ❑ | (6) ❑ |
| JRCC in-charge | (1) ❑ | (2) ❑ | (3) ❑ | (4) ❑ | (5) ❑ | (6) ❑ |
| Responsible ATC | (1) ❑ | (2) ❑ | (3) ❑ | (4) ❑ | (5) ❑ | (6) ❑ |
| Local rescue coordinating central | (1) ❑ | (2) ❑ | (3) ❑ | (4) ❑ | (5) ❑ | (6) ❑ |
| Ambulance services | (1) ❑ | (2) ❑ | (3) ❑ | (4) ❑ | (5) ❑ | (6) ❑ |
| Police on-scene | (1) ❑ | (2) ❑ | (3) ❑ | (4) ❑ | (5) ❑ | (6) ❑ |
| Fire services | (1) ❑ | (2) ❑ | (3) ❑ | (4) ❑ | (5) ❑ | (6) ❑ |
| Defense | (1) ❑ | (2) ❑ | (3) ❑ | (4) ❑ | (5) ❑ | (6) ❑ |
| Voluntary organizations | (1) ❑ | (2) ❑ | (3) ❑ | (4) ❑ | (5) ❑ | (6) ❑ |
| Civil defense | (1) ❑ | (2) ❑ | (3) ❑ | (4) ❑ | (5) ❑ | (6) ❑ |
| Receiving hospital | (1) ❑ | (2) ❑ | (3) ❑ | (4) ❑ | (5) ❑ | (6) ❑ |
| Delivering hospital during Inter-hospital transfer | (1) ❑ | (2) ❑ | (3) ❑ | (4) ❑ | (5) ❑ | (6) ❑ |
| Industry protection services | (1) ❑ | (2) ❑ | (3) ❑ | (4) ❑ | (5) ❑ | (6) ❑ |

Describe what was good / challenging with the cooperation:

________________________________________
________________________________________
________________________________________
________________________________________
________________________________________
________________________________________
________________________________________
________________________________________

In the incident area, how will you describe your knowledge about:

|  | Very bad | Bad | Neither good nor bad | Good | Very good | Unknown |
| --- | --- | --- | --- | --- | --- | --- |
| Hospital | (1) ❑ | (2) ❑ | (3) ❑ | (4) ❑ | (5) ❑ | (6) ❑ |
| Casualty clinic | (1) ❑ | (2) ❑ | (3) ❑ | (4) ❑ | (5) ❑ | (6) ❑ |
| Ambulance service | (1) ❑ | (2) ❑ | (3) ❑ | (4) ❑ | (5) ❑ | (6) ❑ |

| **Cooperation and logistics:** |
| --- |

Who should, in your opinion, coordinate air traffic in uncontrolled airspace with several helicopters during a major incident?

(1) ❑ EMCC

(2) ❑ ATC

(3) ❑ JRCC

(4) ❑ First HEMS or SAR helicopter on-scene

(5) ❑ Other _____

(6) ❑ Unknown

Does guidelines for coordination/cooperation between multiple helicopters on scene in uncontrolled airspace exist in your Company/Squadron?

(1) ❑ Yes

(2) ❑ No

(3) ❑ Unknown

Is the helicopter you operate, in your opinion, equipped with sufficient equipment to allow "situational awareness" in flight safety and other aircrafts?

(1) ❑ Yes

(2) ❑ No

(3) ❑ Unknown

What type of equipment do you miss?

________________________________________________________________________________
________________________________________________________________________________
________________________________________________________________________________
________________________________________________________________________________
________________________________________________________________________________
________________________________________________________________________________
________________________________________________________________________________
________________________________________________________________________________
________________________________________________________________________________
________________________________________________________________________________
________________________________________________________________________________
________________________________________________________________________________
________________________________________________________________________________
________________________________________________________________________________
________________________________________________________________________________

Do you know what VHF flight frequency that is normally used during rescue operations?

(1) ❑ Yes

(2) ❑ No

How do you consider your knowledge about:

|  | Very bad | Bad | Neither good nor bad | Good | Very good | Unknown |
| --- | --- | --- | --- | --- | --- | --- |
| Other rescue services in your catchment area | (1) ❑ | (2) ❑ | (3) ❑ | (4) ❑ | (5) ❑ | (6) ❑ |
| Treatment options at hospitals in your catchment area | (1) ❑ | (2) ❑ | (3) ❑ | (4) ❑ | (5) ❑ | (6) ❑ |
| Other rescue services outside your catchment area | (1) ❑ | (2) ❑ | (3) ❑ | (4) ❑ | (5) ❑ | (6) ❑ |
| Hospital organization outside your catchment area | (1) ❑ | (2) ❑ | (3) ❑ | (4) ❑ | (5) ❑ | (6) ❑ |

| **Competence and procedures:** |
| --- |

Do you have experience in being Medical incident officer (Fagleder helse)?

(1) ❑ Yes

(2) ❑ No

Have you attended a major incident where you, in your opinion, should have been Medical incident officer (Fagleder helse)?

(1) ❑ Yes

(2) ❑ No

Have you attended a major incident where you were Medical incident officer (Fagleder helse), where this was unnecessary?

(1) ❑ Yes

(2) ❑ No

Do you have experience with being Medical on-scene commander (Operativ leder helse)?

(1) ❑ Yes

(2) ❑ No

Have you attended a major incident where you, in your opinion, should have been Medical on-scene commander (Operativ leder helse)?

(1) ❑ Yes

(2) ❑ No

Have you attended a major incident where you were Medical on-scene commander (Operativ leder helse), where this was unnecessary?

(1) ❑ Yes

(2) ❑ No

How do you consider:

|  | Very bad | Bad | Neither good nor bad | Good | Very good | Unknown |
| --- | --- | --- | --- | --- | --- | --- |
| Your competence in organizing a major incident scene? | (1) ❑ | (2) ❑ | (3) ❑ | (4) ❑ | (5) ❑ | (6) ❑ |
| Training you have received in organizing a major incident scene? | (1) ❑ | (2) ❑ | (3) ❑ | (4) ❑ | (5) ❑ | (6) ❑ |
| Your competence in taking the role of Medical incident officer? | (1) ❑ | (2) ❑ | (3) ❑ | (4) ❑ | (5) ❑ | (6) ❑ |
| Training you have received in in taking the role of Medical incident officer? | (1) ❑ | (2) ❑ | (3) ❑ | (4) ❑ | (5) ❑ | (6) ❑ |
| Your competence in taking the role of Medical on-scene commander? | (1) ❑ | (2) ❑ | (3) ❑ | (4) ❑ | (5) ❑ | (6) ❑ |
| Training you have received in in taking the role of Medical on-scene commander? | (1) ❑ | (2) ❑ | (3) ❑ | (4) ❑ | (5) ❑ | (6) ❑ |

Have you participated in the course "Cooperation on-scene" ("Samvirke på skadested")?

(1) ❑ Yes

(2) ❑ No

How many years have gone since you participated on the course?

(1) ❑ 0

(2) ❑ 1

(3) ❑ 2

(4) ❑ 3

(5) ❑ 4 or more

How will you consider your training in handling your allocated tasks in managing a major incident?

(1) ❑ Very bad/Non existent

(2) ❑ Bad

(3) ❑ Neither bad nor good

(4) ❑ Good

(5) ❑ Very good

How many times per year do you train on managing major incidents? (If you work on several bases, please list total amount of training)

(1) ❑ 0

(2) ❑ 1

(3) ❑ 2

(4) ❑ 3

(5) ❑ 4 or more

How often does your service participate in large exercises with other services?

(1) ❑ Every time

(2) ❑ Now and then

(3) ❑ Never

(4) ❑ Unknown

With whom? (Multiple alternatives allowed)

(1) ❑ Police

(2) ❑ Fire

(3) ❑ Ambulance

(4) ❑ Other HEMS/SAR helicopters

(5) ❑ Primary health care

(6) ❑ Rapid response in-hospital teams

(7) ❑ Defense

(8) ❑ Voluntary organizations

(9) ❑ Other _____

On a scale from 1 to 5 where 1 is to little and 5 is to a large extent, will more knowledge and training make you better prepared to manage future major incidents?

(1) ❑ 1

(2) ❑ 2

(3) ❑ 3

(4) ❑ 4

(5) ❑ 5

What do you want more knowledge about/competence in? (Multiple alternatives allowed)

(1) ❑ Leadership

(2) ❑ Decision-making

(3) ❑ Organization

(4) ❑ Communication

(5) ❑ Cooperation with own and other services

(6) ❑ Rescue technical procedures

(7) ❑ Medical procedures/Knowledge

(8) ❑ Triage

(9) ❑ Other _____

(10) ❑ Nothing

Have you been trained in the new guidelines for major incident triage?

(1) ❑ Yes

(2) ❑ No

(3) ❑ Unknown

Have your service available tagging equipment adapted to the new guidelines for major incident triage?

(1) ❑ Yes

(2) ❑ No

(3) ❑ Unknown

What extra equipment for major incident management exists in your service? (Multiple alternatives allowed)

(1) ❑ Communication equipment

(2) ❑ Rescue technical equipment

(3) ❑ Triage equipment

(4) ❑ Stretcher

(6) ❑ Hypothermia preventive equipment

(7) ❑ Medical extra equipment

(8) ❑ Other _____

(9) ❑ Nothing

What extra equipment for major incident management do you miss in your service? (Multiple alternatives allowed)

(1) ❑ Communication equipment

(2) ❑ Rescue technical equipment

(3) ❑ Triage equipment

(4) ❑ Stretcher

(6) ❑ Hypothermia preventive equipment

(7) ❑ Medical extra equipment

(8) ❑ Other _____

(9) ❑ Nothing

| **Finally:** |
| --- |

How many years have you been working in pre-hospital service?

(1) ❑ 0-2

(2) ❑ 2-4

(3) ❑ 4-6

(4) ❑ 6-8

(5) ❑ 8-10

(6) ❑ more than 10 years

Where do you work?

(1) ❑ SAR/Rescue helicopter service

(2) ❑ HEMS

Do you have any suggested improvements in major incident management? What works well/bad?

____________________________________________________________
____________________________________________________________
____________________________________________________________
____________________________________________________________
____________________________________________________________
____________________________________________________________
____________________________________________________________
____________________________________________________________
____________________________________________________________
____________________________________________________________
____________________________________________________________
____________________________________________________________

| *Thank you for taking the time to respond to the survey* |
| --- |
